# The future of epilepsy care in the United Kingdom: A roadmap for technology‐enabled transformation

**DOI:** 10.1002/epi4.70262

**Published:** 2026-04-11

**Authors:** John R. Terry, Rohit Shankar

**Affiliations:** ^1^ Peninsula Medical School University of Plymouth Plymouth UK; ^2^ Neuronostics Ltd Bristol UK; ^3^ Cornwall Partnership NHS Foundation Trust Truro UK

**Keywords:** capable community, equity, prevention, reform, seizures, technology

## Abstract

Epilepsy is recognized to be a significant cause of premature mortality, socio‐economic distress and poor quality of life in economically developed countries. Despite clear clinical guidelines, epilepsy care is marked by delayed diagnosis, fragmented management, high emergency admission rates, and pronounced health inequalities affecting rural populations, ethnic minority groups, and people with intellectual disabilities. Diagnostic pathways remain inefficient, with prolonged waits for electroencephalography (EEG), low sensitivity of routine investigations, and repeated inconclusive testing. Long‐term management continues to depend on infrequent hospital visits and unreliable patient recall, contributing to suboptimal seizure control, avoidable morbidity, and preventable mortality. In the United Kingdom, epilepsy affects over 630 000 people, accounts for approximately £2 billion in annual healthcare costs and exemplifies these systemic failures. Current hospital‐centric care models are failing to meet their complex, often lifelong, needs. In its recent 2025 “Fit for the Future” 10‐year plan, the UK government mandates three fundamental shifts in healthcare: *from hospital to community*, *analogue to digital*, and *sickness to prevention*. Epilepsy diagnosis and care exemplify the potential for this comprehensive triumvirate transformation, with emerging technologies including point‐of‐care EEG systems, AI‐powered diagnostics, wearable devices, and digital therapeutics offering unprecedented opportunities to deliver specialist‐level care in community settings and reduce illness burden. However, successful implementation requires addressing digital exclusion risks for vulnerable populations. We propose that technology‐enabled community epilepsy care can serve as a blueprint for the UK's National Health Service (NHS) transformation while delivering immediate benefits for patients, families, and healthcare systems. The convergence of clinical need, technological capability, and policy imperative creates a unique opportunity to move beyond incremental improvements to fundamental system redesign that ensures equitable access to high‐quality epilepsy care across all communities. Such a model, if delivered, could be an exemplar for other chronic conditions both in the United Kingdom and globally.

**Plain Language Summary:**

Current epilepsy care in the United Kingdom often involves long waits and relies on infrequent hospital visits, which is not ideal for a lifelong condition. New technologies, such as wearable devices and AI‐powered tools, offer a chance to change this. By moving care from hospitals into the community, we can provide faster diagnosis, continuous monitoring, and more personalised support. This will help improve the lives of the 630 000 people with epilepsy in the UK, ensuring they receive better, more accessible, and more equitable care.

**Key points**

- Epilepsy exemplifies the UK NHS crisis, with delayed diagnosis, fragmented care, and marked health inequalities.
- Hospital‐centric epilepsy pathways drive poor outcomes, high emergency admissions, and avoidable costs.
- Community‐based technologies can enable earlier diagnosis, continuous monitoring, and preventive care.
- AI, wearables, and digital tools offer specialist‐level insights beyond traditional clinic settings.
- Equitable implementation is essential to ensure innovation reduces, not widens, epilepsy inequalities.

## 1 INTRODUCTION

The NHS in England in 2025 stands at a crossroads. Lord Darzi's independent investigation delivered a stark verdict: the health service is in “critical condition,” with over 58% of the NHS' £200 Billion annual budget concentrated in hospitals and insufficient investment in community care.^1^ For people with epilepsy, this issue has been previously recognized The hospital‐centric model has been the historic cause of access barriers, delayed diagnoses, and fragmented care that exemplifies the broader systemic failures Lord Darzi identifies.

**FIGURE 1:**
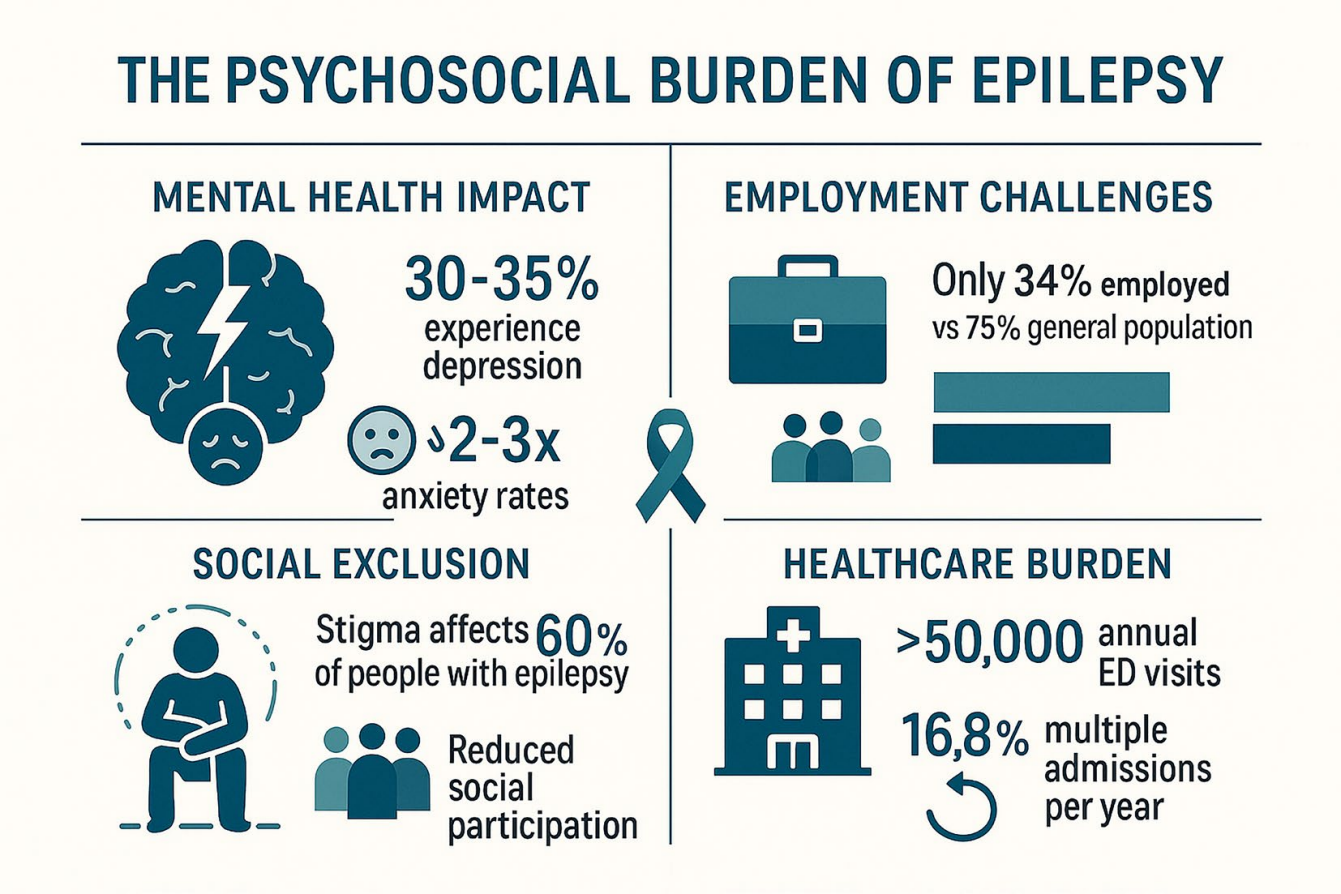
Psychosocial burden of epilepsy.

Epilepsy affects 630 000 people in the United Kingdom,^2^ causing three to five deaths every day and costing the NHS around £2 billion annually, that is, roughly 1% of the entire NHS budget.^3^, ^4^, ^5^ Despite this substantial burden, epilepsy care remains characterized by significant inequalities, with rural communities, ethnic minorities, and people with intellectual disabilities experiencing particularly poor outcomes.^6^, ^7^ The psycho‐social burden of epilepsy is outlined in Figure 1. The unpredictable nature of the condition, frequent and often complex comorbidities, lifelong management requirements, and premature mortality for a significant minority make epilepsy an ideal test case for the fundamental transformation that the NHS requires to survive and thrive.^8^

In their response to the Darzi Review, “Fit for the Future: 10 Year Health Plan for England,” the Government mandates three strategic shifts that directly address epilepsy care challenges: f*rom hospital to community*, *analogue to digital*, and *sickness to prevention*.^9^ These shifts align well with the research priorities identified by the 2024 James Lind Alliance Priority Setting Partnership for Epilepsy, which engaged over 5000 people affected by epilepsy to establish consensus research priorities.^10^ The convergence of policy imperative, technological capability, and community‐identified needs creates an unprecedented opportunity for transformative change, as well as future research leading to potential wealth creation for the country.

## 2 CURRENT EPILEPSY CARE: A CRISIS WITHIN A CRISIS

The current epilepsy care pathway epitomizes the inefficiencies and inequalities that characterize the broader NHS crisis. Following a suspected seizure, patients face diagnostic delays that would be unacceptable in other areas of medicine. NICE quality standards recommend an electroencephalogram (EEG) be performed as quickly as possible post event (ideally within 72 h) with assessment by someone with epilepsy specialism within two weeks.^4^ However, at present, more than half of people with suspected epilepsy are waiting longer than six weeks for an EEG, with times over eight months not uncommon.^6^, ^11^

The diagnostic challenge is compounded by the reduced ability of routine EEGs to correctly identify epilepsy when it is present, that is, low sensitivity. Studies have consistently found sensitivity to be lower than 20%.^12^, ^13^ Furthermore, since epilepsy is only one cause of presenting with suspected seizures, the overall rate of noncontributory (inconclusive) EEGs approaches 70–80% in clinical practice.^14^ This creates a vicious cycle of delayed access to EEG, with the prolonged time interval of access lowering EEG's diagnostic yield, in turn necessitating repeat investigations and perpetuating further delays.

Once a confirmed diagnosis is finally made, ongoing management relies heavily on infrequent hospital consultations that depend on patient recall of seizure activity, medication side effects, and quality of life impacts. This data may be incomplete or inaccurate due to postictal confusion, memory impairment, or lack of compliance. As a result, while around half of people with a new diagnosis of epilepsy will become seizure‐free on their first anti‐seizure medication, a significant challenge remains: over time, around one‐third do not respond appropriately to any combination of anti‐seizure medications.^15^ Suspected seizures represent the most common neurological cause of unscheduled admissions to A&E, with studies suggesting >50 000 such admissions annually.^16^ With 16.8% of patients experiencing multiple admissions per year, this pattern reflects failures of the current approach to managing seizure disorders.

However, seizure control, while an important indicator of premature mortality,^17^ is not the only one. A broader analysis of emergency department presentations reveals that repeat attendances are frequently driven by factors beyond seizure activity: alcohol and substance misuse, mental illness, social deprivation, and homelessness.^18^ This pattern creates a risk of major disenfranchisement from pathways of inclusion, where people with epilepsy become trapped in cycles of crisis‐driven care that fail to address underlying social determinants.

The consequent impact on health inequalities is profound.^8^ Rural communities face additional barriers due to travel distances and limited access to clinicians with epilepsy specialism.^19^ Ethnic minority populations experience delayed diagnosis and suboptimal treatment outcomes,^20^ while people with intellectual disability who have significantly higher epilepsy prevalence often receive fragmented care that fails to address their complex needs.^7^, ^21^

The economic implications are equally stark. The £2 billion annual cost of epilepsy care could be substantially reduced through more effective community‐based person‐centered, holistic management, yet current funding models incentivize hospital‐based interventions rather than preventive community care. This misalignment of incentives perpetuates a reactive model that treats seizures after they occur rather than preventing them through proactive monitoring and intervention.^18^

## 3 INTERPRETING DARZI'S VISION IN THE CONTEXT OF EPILEPSY CARE: THE CASE FOR EMERGING TECHNOLOGIES

The Darzi Review calls for a “tilt towards technology” to unlock productivity gains. This is particularly relevant for epilepsy care, where technological solutions can address fundamental limitations of traditional hospital‐based models. The three strategic shifts proposed by the government in response provide a framework for epilepsy care transformation that addresses both immediate needs as well as long‐term sustainability. Moving from *hospital to community* could be enabled by faster access to diagnostic tests, more regular monitoring, as well as ongoing management in the natural environment of the person, particularly in those with complex needs such as intellectual disability.^20^ This would provide more complete data, while reducing travel burdens and access barriers. The shift from *analogue to digital* may be exemplified by transforming seizure tracking from unreliable patient recall to more objective, continuous monitoring that enables faster clinical decision‐making. Predictive algorithms offer the potential to move from *an illness to prevention model* enabling proactive interventions rather than reactive responses in the aftermath of suspected seizures.

Enabling these changes would directly address community‐identified priorities.^9^ These emphasized a desire for tools and devices to accurately predict seizures and the use of data and AI to aid the diagnosis and management of epilepsy. Technology‐enabled care offers unprecedented opportunities to identify and address these complex needs through sophisticated risk stratification. AI‐powered risk scores can identify high‐risk individuals who would benefit from enhanced social support and wraparound services. By integrating social determinants data with clinical indicators, predictive algorithms can enable proactive intervention before crisis points are reached.

Technology platforms could facilitate seamless integration between medical management, mental health support, social services, and community resources, creating comprehensive care ecosystems that address the full spectrum of epilepsy‐related needs. Such a holistic approach would ensure *improvements in quality of life*, as well as ultimately, the *prevention of deaths due to epilepsy*. The economic potential of technology‐enabled epilepsy care is substantial, with reduced hospital admissions, improved medication adherence, and better seizure control translating to significant cost savings while improving patient outcomes and quality of life.

## 4 IMPLEMENTATION CHALLENGES AND EQUITY CONSIDERATIONS

The successful adoption and implementation of technology‐enabled epilepsy care is dependent on several factors. These include:

1. *Regulatory Status*: The regulatory approval status (e.g., FDA‐cleared, UKCA‐marked) and the strength of the evidence supporting the approval(s) of the technology.
2. *Clinical Evidence Quality*: The quality and quantity of the clinical evidence supporting the safety and effectiveness of the technology, with a focus on peer‐reviewed studies and meta‐analyses.
3. *Real‐World Performance*: The performance of the technology in real‐world settings, including its accuracy, reliability, and usability outside of controlled research environments.
4. *Population Suitability*: The suitability of the technology for different patient populations, taking into account factors such as age, ethnicity, and comorbidities.
5. *Implementation Feasibility*: The feasibility of implementing the technology in clinical practice, including the need for training, infrastructure, and integration with existing workflows.
6. *User Acceptability*: The acceptability of the technology to patients and caregivers, including factors such as comfort, convenience, as well as potential burdens, such as false alarms.

## 5 COMMUNITY‐BASED TECHNOLOGY SOLUTIONS TO REDUCE HOSPITAL INVOLVEMENT

We highlight six areas where technology could enable a shift from hospital to community‐based care in the coming years and play a role in optimized diagnosis and management. For each area, we aim to highlight a range of solutions and maturity levels. These range from *mature*: by which we mean technologies with some form of regulatory approval (FDA/MHRA/CE), established clinical evidence, and current deployment in healthcare settings, *approaching maturity*: those with regulatory approval and clinical validation but requiring quality assurance protocols and training programs for widespread deployment, to *developing*: emerging technologies in clinical development or early deployment phases. A summary of all technologies presented in the subsequent sections is presented in Table 1.

**TABLE 1:** Details of major technologies discussed.

Section	Company (product)	Website
1. Point‐of‐Care EEG	Zeto (Zeto Headset)	https://zeto‐inc.com/
	Neuroelectrics (Enobio system)	https://www.neuroelectrics.com/
	BrainCapture (BC‐1 system)	https://www.braincapture.dk/
	BitBrain	https://www.bitbrain.com/
	BrainScope	https://www.brainscope.com/
	Ceribell (Rapid Response EEG)	https://ceribell.com/
2. Long‐Term Brain Monitoring	UNEEG (EpiSight)	https://www.uneeg.com/
	EpiMinder (Minder)	https://epiminder.com/
	Beacon Biosignals (DREEM 3S system)	https://beacon.bio/dreem‐3s
	Eysz	https://eyszlab.com/
	Epihunter	https://www.epihunter.com/
	Hurulabs	https://www.hurulabs.com/
3. AI‐enabled Analytics	Holberg EEG/Natus (SCORE‐AI)	https://www.holbergeeg.com/score‐ai
	Massachusetts General Hospital (SpikeNet)	https://advances.massgeneral.org/neuro/journal.aspx?id=1451
	Neuronostics (BioEP platform)	https://www.neuronostics.com/
	Deegtal	https://www.deegtal.ai/
	Neurologic Solutions	https://neurologicsolutions.net/
	Piramidal	https://piramidal.ai/
4. Wearable Devices	Empatica (Embrace2)	https://www.empatica.com/
5. Digital Apps	SUDEP Action (EpSMon)	https://sudep.org/about‐research/epsmon‐app/
6. Telemedicine	No specific companies	N/A

### 5.1 Point‐of‐Care EEG: bringing neurophysiology to primary care

Point‐of‐care (PoC) EEG systems represent a cornerstone technology for shifting epilepsy diagnosis from specialized hospital settings into the community. These devices are easy‐to‐apply systems, using dry or wet electrodes. In most cases they require limited training to apply, making them ideal candidates for use in community settings with the potential to fundamentally reshape the diagnostic journey for people who have experienced a suspected seizure.

#### 5.1.1 Current evidence levels

Examples of such systems include the Zeto Headset, an FDA‐approved system that offers a full montage wireless, dry‐electrode EEG acquisition that requires no gel application or extensive scalp preparation. Clinical studies demonstrate diagnostic concordance with traditional EEG while enabling acquisition by nonspecialist healthcare providers.^22^ Similarly, the Enobio system from Neuroelectrics is a CE‐ and FDA‐approved device offering 20‐ and 32‐channel configurations in both dry‐ and gel‐based electrode options for flexible clinical deployment. The BrainCapture BC‐1 system offers 10–20 EEG using an EEG cap with gel electrodes. Designed for use in remote, rural locations in Africa and Indonesia, the system can be deployed following as little as 30 min training.^23^ The field is evolving rapidly with BitBrain and BrainScope offering FDA‐cleared devices for simplified EEG acquisition. Equally, established systems such as the Ceribell rapid response EEG, designed for use in emergency rooms, offer the potential to be repurposed for community‐based use. These systems typically offer Bluetooth and Wi‐Fi enabled communication with cloud‐based analytics, a further advantage in community settings where traditional infrastructure may be unavailable.

#### 5.1.2 Challenges and limitations

Despite their promise, the widespread adoption of PoC EEG systems faces several challenges. The evidence base, while growing, is still limited in terms of real‐world diagnostic accuracy and cost‐effectiveness studies in community settings. A key concern is the potential for misinterpretation of EEG data by nonspecialists, which could lead to both over‐ and under‐diagnosis of epilepsy. The need for robust quality assurance protocols, standardized training programs, and clear referral pathways are all critical barriers that must be addressed before PoC EEG can be safely and effectively integrated into routine clinical practice. Additionally, the cost‐effectiveness of these systems compared to traditional EEG pathways remains to be established through detailed health economic analyses.

### 5.2 Long‐term brain monitoring: enabling continuous surveillance

A key challenge in epilepsy is that seizures are typically infrequent and seemingly unpredictable, occurring most commonly outside of clinical settings. The ability to monitor the brain long‐term represents another opportunity for community‐based epilepsy care. Such systems could enable better characterization of seizure type, capture the true seizure burden, and ultimately may inform the development of personalized seizure forecasting algorithms.

#### 5.2.1 Current evidence levels

Long‐term monitoring systems are already showing considerable promise in clinical studies. Subcutaneous systems such as EpiSight from UNEEG or Minder from EpiMinder present CE‐marked and FDA‐approved implantable systems that can monitor subscalp EEG continuously over months, or even years. EpiSight has demonstrated clinical potential in drug‐resistant epilepsy,^24^ while the UMPIRE study^25^ has demonstrated the effectiveness of the Minder system's bilateral subscalp system for seizure detection. These devices provide signal quality equivalent to traditional scalp EEG systems but with lower noise levels and no requirement for electrode maintenance.

Scalp systems, such as the DREEM 3S system, have been validated for overnight EEG monitoring with FDA‐approved PSG‐quality signal acquisition, offering a noninvasive option for nocturnal seizure monitoring.^26^ For many people with epilepsy, whose seizures occur predominantly, or even exclusively, at night, this system has the potential to capture seizure activity that would otherwise go undetected, providing clinicians with more accurate data on seizure burden and patterns. Other devices such as Epihunter: a lightweight EEG headband providing continuous monitoring specifically optimized for absence seizures. When paired with a smartphone, the system triggers automated video recording upon detection of potential seizure activity to enable more objective documentation.^27^ Eysz has created sensor dots that enable monitoring of EEG‐like signals for extended periods using adhesive sensors. The portability of the system and the improved comfort when worn for long periods make the solution potentially suitable for long‐term community use.

Alongside these established systems there are several emerging technologies that may offer further benefits. For example, Hurulabs is developing a novel EEG biosensor technology that aims to provide clinical‐grade signal quality but in a highly discreet manner. This may help reduce the stigma many people with epilepsy feel when wearing devices, making it potentially suitable for long‐term monitoring in the community.

#### 5.2.2 Challenges and limitations

Long‐term monitoring systems face significant implementation challenges. The invasive nature of subcutaneous systems like EpiSight requires surgical implantation, limiting their applicability to selected patient populations and requiring specialist surgical expertise. Real‐world studies suggest that while these systems can provide valuable clinical information, patient selection criteria remain unclear, and the clinical impact on treatment decisions requires further validation. For scalp‐based systems, comfort and tolerability during extended wear periods can be problematic, with some people experiencing skin irritation or discomfort that limits compliance. A further challenge is the cost of many of these systems and the need for data interpretation by trained specialists also present barriers to widespread adoption. Furthermore, the integration of continuous monitoring data into existing clinical workflows and electronic health records remains a significant technical challenge. Standardized reporting formats and clinical guidelines will be critical for widespread adoption.

### 5.3 AI‐enabled analytics: making sense of big data

Bringing EEG to point‐of‐care for diagnosis, alongside long‐term EEG monitoring for ongoing management will create substantial volumes of data that existing systems and approaches to EEG interpretation will be unable to cope with. Here, emerging AI solutions offer the potential to prescreen data and so offer the potential to enable first EEGs to occur within community settings, with cases triaged for further assessment by trained experts.

#### 5.3.1 Current evidence levels

AI‐powered EEG interpretation has demonstrated impressive performance in clinical studies. The SCORE‐AI system from Holberg EEG (recently acquired by Natus) has been shown to achieve diagnostic accuracy equivalent to that of trained neurophysiologists, with a large‐scale validation study demonstrating its potential to democratize access to specialist‐level expertise.^28^ The system uses advanced machine learning algorithms trained on a database of over 30 000 recordings and has been validated in external settings.^29^ A similar approach using a deep neural network trained on almost 10 000 recordings (SpikeNet) has been developed by Massachusetts General Hospital and also achieved levels of performance equivalent to trained neurophysiologists.^30^ In both cases, the systems appear particularly suited to identifying EEGs that are genuinely free from traditional interictal epileptiform abnormalities. Alongside on‐board tools provided by Beacon Biosignals, Ceribell, EpiMinder, and UNEEG, these automated analysis tools offer the potential to reduce the significant volumes of data generated to enable focus on specific patterns of concern.

A further key challenge with the use of EEG is that over 70% of routine EEGs are currently inconclusive. An apparently normal EEG neither excludes a diagnosis of epilepsy nor confirms an alternative condition. Here tools such as Neuronostics' BioEP platform may play an important role. BioEP is a UKCA‐marked software that uses eight features of the EEG that are known to be altered in people with epilepsy. It gives a rating of the level of support for epilepsy being the cause of symptoms from apparently normal EEG. In published research, the technology has been shown to achieve levels of sensitivity in excess of what can be achieved using EEG to detect traditional interictal epileptiform abnormalities.^31^ For inconclusive EEGs collected in the community, this may offer a powerful tool for risk stratification, identifying people with a high likelihood of epilepsy who can then be prioritized for specialist follow‐up.^32^ There are further emerging tools from companies such as Deegtal, Neurologic Solutions, or Piramidal that use deep‐learning algorithms to recognize subtle patterns that may indicate the presence of epilepsy from EEG that would otherwise be considered clinically normal.^33^

#### 5.3.2 Challenges and limitations

While the evidence for AI‐enabled EEG analytics is promising, several challenges remain. Many of the emerging platforms lack robust, independent validation, and there is a need for more real‐world studies to assess their clinical utility and impact on patient outcomes. Many AI systems are trained on too few examples relative to the number of patterns they're trying to detect, like trying to learn a language from just a handful of sentences. The rule of thumb is at least 15 examples for every pattern the algorithm is learning to detect. Many emerging systems fall short of this threshold, raising concerns that impressive research results may in fact be due to overfitting.^34^, ^35^ This means that promising research results will not translate into real‐world utility. Additionally, the “black box” nature of most deep learning algorithms can also be a barrier to clinical adoption, with clinicians often reluctant to trust the outputs of systems they do not fully understand. The integration of these systems into existing clinical workflows also presents a significant technical and logistical challenge, requiring substantial infrastructure investment and staff training.

### 5.4 Wearable devices: tools for seizure detection

Wearable seizure detection devices are a maturing technology naturally suited to use in the community. Such devices may address the fundamental limitation of hospital‐based monitoring by providing continuous home‐based assessment. They may facilitate immediate caregiver alerts or provide objective documentation of seizure timings that inform ongoing clinical decision‐making.

#### 5.4.1 Current evidence levels

A growing body of evidence supports the use of wearable devices for seizure detection. A meta‐analysis of 23 studies (1269 patients, median recording 52.9 h) found that noninvasive wearables achieved a mean sensitivity of 0.91 for the detection of tonic–clonic seizures, with wrist‐worn devices demonstrating particularly high accuracy.^36^ The Empatica Embrace2 has been shown to detect generalized tonic–clonic seizures with high sensitivity in controlled settings, leading to its FDA clearance for this indication.^37^ Surface EMG devices have demonstrated 93.8% sensitivity for tonic–clonic seizure detection with a median detection latency of nine seconds.^38^ For absence seizures, wearable EEG devices have achieved sensitivity rates of 78.8% with many patients experiencing no false alarms.^27^ These devices have the potential to significantly improve the safety and quality of life for people with epilepsy, particularly those who are at high risk of seizure‐related injury or SUDEP.

#### 5.4.2 Challenges and limitations

Despite their high sensitivity for tonic–clonic seizures, wearable devices have significant limitations that affect their real‐world utility. The most significant of these is the high rate of false alarms, with studies showing that only approximately 15% of alerts from devices like the Empatica Embrace2 represent a true seizure.^26^ These false alarms, which can be triggered by everyday activities such as teeth brushing, cycling, and food preparation, can cause significant anxiety for patients and their families, and may lead to alarm fatigue and nonadherence. The false alarm rate is particularly problematic in pediatric populations, where rates can be twice as high as in adults.^36^ The performance of wearable devices is also less impressive for non‐motor seizures, with limited evidence for detection of focal seizures without motor manifestations. The cost of these devices can also be a barrier to access, and there is a need for clear commissioning pathways to ensure that they are available to all who could benefit. User acceptability studies suggest that while many patients value the safety benefits, the burden of false alarms can significantly impact quality of life to the extent that device adherence becomes an issue.

### 5.5 Digital apps: empowering self‐management

Patient‐used technology in epilepsy now spans wearables for real‐time seizure detection, mobile apps and electronic seizure diaries, at‐home/portable EEG systems, and seizure‐risk forecasting tools all aiming to make seizure documentation more objective, improve safety, and enable personalized management between clinic visits.

#### 5.5.1 Current evidence levels

Digital seizure diaries have shown promise in improving the quality and quantity of seizure documentation. A review of nine electronic diaries showed that despite feature differences between the diaries, overall patients could log seizures quickly and accurately, thus improving the quantity and quality of data, which can be used for clinical and research benefit.^39^ This was further supported by a study which suggested that those reporting seizures in an app demonstrated better precision than those using paper records, with app‐based reporting achieving 85.7% precision compared to 66.9% for paper diaries.^40^, ^41^ EpSMon (Epilepsy Safety Monitor), the digital version of the validated SUDEP & Seizure Safety Checklist, has gained significant traction with over 5000 registered users and recognition by the NHS RightCare toolkit and the Neurological Alliance. The app allows people with epilepsy to record and compare their change in risk factors on a three‐monthly basis, providing personalized reports for healthcare professionals.

#### 5.5.2 Challenges and limitations

Despite their potential benefits, digital apps face significant challenges that limit their clinical impact. A major limitation is that seizure diaries, whether digital or paper‐based, achieve only moderate sensitivity of approximately 50%, meaning that half of seizures go undocumented, regardless of the recording method.^42^, ^43^ A systematic review of 22 self‐management apps in epilepsy found that installation rates were generally low, and there was no strong evidence of their effectiveness in improving seizure control or quality of life.^44^ The digital divide also presents significant equity concerns, as app effectiveness may be influenced by factors such as education levels, technology comfort, social class, as well as ethnicity, potentially creating bias in user populations. Furthermore, most apps lack robust clinical validation, and there is limited evidence of their impact on clinical outcomes or healthcare utilization.

### 5.6 Telemedicine: Barrier‐Free Access to Specialist Expertise

Telemedicine represents a mature technology ideally suited for enabling specialist epilepsy consultations to reach underserved communities while reducing travel burdens and access barriers. The integration of telemedicine with remote monitoring technologies creates comprehensive care ecosystems where specialists located anywhere in the world can review real‐time data from wearable devices, seizure diaries, and point‐of‐care EEG systems and subsequently provide virtual consultations.

#### 5.6.1 Current evidence levels

Telemedicine has demonstrated effectiveness in epilepsy care, particularly during the COVID‐19 pandemic when remote consultations became essential. Studies have shown that telemedicine consultations can maintain the quality of care while improving access for patients in rural or underserved areas. The technology enables specialist expertise to reach communities that would otherwise have limited access to epilepsy specialists, addressing geographic inequalities in care. Integration with remote monitoring technologies allows for a more comprehensive assessment of seizure patterns and medication effects between clinic visits. Telemedicine platforms can also facilitate multidisciplinary team meetings and case discussions, improving coordination of care across different healthcare providers.

#### 5.6.2 Challenges and limitations

Despite its maturity, telemedicine faces several implementation challenges in epilepsy care. The digital divide affects access, with some patient populations lacking reliable internet connectivity or the technical skills needed to use telemedicine platforms effectively. Clinical examination limitations mean that certain aspects of neurological assessment may be compromised in virtual consultations. There are also concerns about the quality of seizure history taking in virtual settings, where nonverbal cues and family dynamics may be harder to assess. There are also user concerns about data security and privacy in virtual care platforms.

In Table 2, we present our summary of the current state of each technology type across these six key areas.

**TABLE 2:** Summarizing the implementation readiness of different technologies.

Technology	Regulatory status	Clinical evidence	Real‐world performance	Population suitability	Implementation feasibility	User acceptability	NHS priority
PoC EEG	CE/FDA‐ approved	Strong technical validation	Limited community data	Hair texture concerns	Training requirements	Generally positive	High
Long‐Term Monitoring	CE/FDA‐ approved	Growing evidence	Patient selection unclear	Invasive procedures	Specialist requirements	Comfort limitations	Medium
AI Analytics	Variable approvals	Strong peer‐reviewed	Generalizability questions	Demographic bias risk	Workflow integration	Clinician acceptance	High
Wearables	FDA‐cleared (limited)	Strong meta‐analysis	High false alarm burden	Age‐dependent performance	Cost barriers	Mixed due to alarms	Medium
Digital Apps	Limited regulation	Moderate evidence	High dropout rates	Digital divide concerns	Low technical barriers	Variable engagement	Medium
Telemedicine	Established	Proven effectiveness	Infrastructure‐dependent	Digital divide	Mature platforms	Generally positive	Medium

## 6 BROADER FACTORS

Beyond these core factors, careful attention to digital exclusion risks that could exacerbate existing health inequalities is needed. For example, ethnic minority populations face multiple barriers to technology adoption, including language barriers and cultural concerns about data sharing and digital use. Implementation strategies must include culturally appropriate training programs, multilingual interfaces, and community engagement initiatives that build trust and understanding. The involvement of community leaders and culturally aware healthcare providers is essential for successful technology adoption in diverse communities.

Likewise, people with intellectual disabilities will require specific adaptations to ensure technology accessibility. User interfaces must accommodate cognitive differences through simplified navigation, visual cues, and alternative communication methods. Carer involvement and support systems are essential, while maintaining individual autonomy and choice wherever possible.

Rural communities face unique challenges. These include limited mobile connectivity, poor broadband speeds, reduced availability of technical support, alongside greater distances to access appropriate services. Implementation strategies must include infrastructure investment, local training programs, and robust technical support systems that ensure reliable technology access regardless of geographic location.

In general, a shift to digital‐first healthcare risks excluding those who lack access to technology, digital skills, or reliable internet connectivity. Implementation must include alternative access pathways, digital inclusion initiatives, and support for technology acquisition and training. The principle of “digital by default, not digital only” ensures that technology enhances rather than replaces traditional care pathways.

## 7 ECONOMIC CASE AND RESOURCE ALLOCATION

The economic case for technology‐enabled epilepsy care extends far beyond immediate healthcare cost savings to encompass three critical dimensions: direct healthcare savings, income generation opportunities, and environmental sustainability.

### 7.1 Healthcare cost reduction

The £2 billion annual cost of epilepsy care could be substantially reduced through technology‐enabled prevention and community management. Emergency admissions alone account for over £150 million annually, with >50 000 seizure‐related presentations.^16^ Improved seizure control and quality of life enable better employment outcomes, with only 34% of people with epilepsy currently in employment compared to 75% in the general population.^45^

### 7.2 Income generation and commercial potential

The commercialization of epilepsy technologies presents significant opportunities for UK PLC, aligning with the O'Shaughnessy Review's emphasis on clinical trials and life sciences innovation.^46^ The global epilepsy device market is projected to grow by over 50% to >£2B by 2032, with the United Kingdom well‐positioned to capture market share through its world‐class research base and NHS testbed capabilities.

Following the successful integration of technology to manage diabetes, UK‐developed epilepsy innovations could generate substantial export revenue while establishing the country as a global leader in technology for neurological diagnosis and care. The integration of university research, NHS validation, and commercial development creates a powerful ecosystem that drives innovation and delivers both clinical and economic benefits.

### 7.3 Environmental sustainability

Hospital admissions account for 40% of NHS carbon emissions, making community‐based care a critical component of the NHS Net Zero strategy. Technology‐enabled epilepsy care can significantly reduce carbon footprint through decreased hospital utilization (especially emergency department admissions), reduced travel for consultations, and improved medication adherence that minimizes wastage.

Medication noncompliance in epilepsy leads to substantial pharmaceutical waste, with environmental implications beyond direct costs. Digital therapeutics and remote monitoring could improve adherence rates while reducing the environmental impact of unused medications and their disposal.

## 8 CONCLUSION AND FUTURE DIRECTIONS

The transformation of epilepsy care through technology‐enabled community solutions offers a compelling blueprint for broader NHS reform. The convergence of clinical need, technological capability, and policy imperative creates a unique opportunity to move beyond incremental improvements to fundamental system redesign. Addressing the systemic challenges identified in the Darzi Review will ultimately deliver benefits for patients, families, and healthcare systems.

The three proposed strategic shifts: *hospital to community, analogue to digital*, and *sickness to prevention* find perfect expression in a transformed epilepsy care pathway. Point‐of‐care EEG systems bring specialist diagnostics to community settings, wearable devices enable continuous monitoring in natural environments, AI‐powered analytics provide specialist‐level insights regardless of geographic location, and digital apps empower patient self‐management while reducing healthcare utilization.

Ultimately, the successful integration of these technologies requires a strategic and coordinated approach. The NHS, with its unique combination of rich data assets and world‐leading research infrastructure, is perfectly positioned to lead the way in generating the evidence needed to unlock the full potential of these transformative technologies. To achieve this, we propose a roadmap with the following three key pillars:

1. *Strategic Evidence Development*: The NHS should establish a national epilepsy technology research program to coordinate and fund the clinical validation of promising new technologies. This program should prioritize:
  - Real‐world validation studies: To assess the performance of technologies in diverse populations and clinical settings
  - Health economic analyses: To quantify the cost‐effectiveness of new technologies and inform commissioning decisions.
  - Implementation studies: To identify the barriers and facilitators to the successful adoption of new technologies in clinical practice.
2. *A Supportive Regulatory and Commissioning Environment*: The NHS should work with regulatory bodies, such as the MHRA, to create a clear and streamlined pathway for the approval of new epilepsy technologies. It should also develop innovative commissioning models that incentivize the adoption of evidence‐based technologies and support the collection of real‐world data
3. *A National Implementation Strategy*: The NHS should develop a national implementation strategy for epilepsy technology, with clear guidance on which technologies should be adopted, in which patient populations, and at what stage of the care pathway. This strategy should be developed in collaboration with clinicians, researchers, patients, and industry partners, and it should be supported by a comprehensive program of education and training.

The economic case for transformation is compelling, with substantial potential for healthcare cost reductions alongside improved patient outcomes and quality of life through reduced hospital admissions, better medication adherence, and enhanced seizure control. More importantly, technology‐enabled epilepsy care demonstrates that the NHS can embrace innovation while maintaining its founding principles of universal care, free at the point of delivery, based on need rather than ability to pay.

However, beyond a simple roadmap, successful transformation requires additional considerations. For example, the workforce training and development required to equip healthcare professionals with the skills to use and interpret these new technologies effectively. Additionally, the intentional inclusion of vulnerable populations to ensure that technology enhances rather than exacerbates existing health inequalities. The experiences of ethnic minorities, people with intellectual disabilities, and rural communities must inform implementation strategies that prioritize equity alongside innovation.

The 630 000 people with epilepsy in the United Kingdom deserve better than the current hospital‐centric model that fails to meet their complex, lifelong needs. They deserve a healthcare system that harnesses the power of technology to deliver specialist‐level care in their communities, prevents seizures before they occur, and empowers them to take control of their condition. The time for incremental change has passed; the moment for transformation is now.

## AUTHOR CONTRIBUTIONS

JT—Conceptualization, Formal analysis, Funding Acquisition, Investigation, Methodology, Project administration, Validation, Visualization, Writing—original draft, Writing—review and editing. RS—Conceptualization, Formal analysis, Investigation, Methodology, Project administration, Validation, Visualization, Writing—review and editing. Both authors satisfy the ICMJE guidance by substantially contributing to the design, analysis and interpretation of the work, drafting of the manuscript, final approval of the manuscript and all agree to be accountable for all aspects of the work in ensuring that questions related to the accuracy or integrity of any part of the work are appropriately investigated and resolved.

## FUNDING INFORMATION

JRT acknowledges the financial support of EPSRC via grant EP/T027703/2. Both authors acknowledge the financial support of EPSRC via grant EP/W035030/1.

## CONFLICT OF INTEREST STATEMENT

There is no direct disclosure or conflict of interest for any author for this submitted body of work. JRT is a cofounder and managing director of Neuronostics Ltd and a Theme Lead for enabling technologies at the Epilepsy Research Institute. RS developed the noncommercial and free‐to‐use SUDEP and Seizure Safety Checklist and the EpSMon app to reduce the risk of SUDEP and enhance seizure safety. RS is the chief investigator of the NIHR‐adopted national Ep‐ID register. The Register is supported and monitored by the National Institute of Health Research, UK. The funding for each molecule examined by the Register is via an Investigator Initiated Support grant from each of the molecule's parent companies. The funding is provided to RS's NHS institution and goes toward the salary of the research coordinator and the institution's project oversight costs. The contributing companies till date include Eisai, UCB, Bial, Jazz Pharma (previously GW Pharma) and Angelini. This work sits outside the submitted work. In addition to the above, RS has received institutional research, travel support and/or honorarium for talks and expert advisory boards from LivaNova, UCB, Eisai, Neuraxpharm, Veriton Pharma, Bial, Angelini, UnEEG and Jazz/GW Pharma outside the submitted work. He holds or has held competitive grants from various national grant bodies, including Innovate, Economic and Social Research Council (ESRC), Engineering and Physical Sciences Research Council (ESPRC), National Institute of Health Research (NIHR), NHS Small Business Research Initiative (SBRI) and other funding bodies, including charities, all outside this work. No other author has any declared conflict of interest related to this paper. We confirm that we have read the journal's position on issues involved in ethical publication and affirm that this report is consistent with those guidelines.

## Data Availability

Data sharing is not applicable to this article as no new data were created or analyzed in this study.
